# Origin of Activity and Stability Enhancement for Ag_3_PO_4_ Photocatalyst after Calcination

**DOI:** 10.3390/ma9120968

**Published:** 2016-11-29

**Authors:** Pengyu Dong, Guihua Hou, Chao Liu, Xinjiang Zhang, Hao Tian, Fenghua Xu, Xinguo Xi, Rong Shao

**Affiliations:** 1 Key Laboratory for Advanced Technology in Environmental Protection of Jiangsu Province, Yancheng Institute of Technology, Yancheng 224051, China; houguihua@ycit.cn; 2 School of Materials Engineering, Yancheng Institute of Technology, Yancheng 224051, China; cliu@ycit.cn (C.L.); zhangxj1983@yahoo.com (X.Z.); tianhao729@yahoo.com (H.T.); xfhuachina@sina.com (F.X.); 3 Jiangsu Collaborative Innovation Center for Ecological Building Materials and Environmental Protection Equipments, Yancheng Institute of Technology, Yancheng 224051, China; xxg@ycit.cn

**Keywords:** Ag_3_PO_4_, photocatalysis, oxygen vacancies

## Abstract

Pristine Ag_3_PO_4_ microspheres were synthesized by a co-precipitation method, followed by being calcined at different temperatures to obtain a series of calcined Ag_3_PO_4_ photocatalysts. This work aims to investigate the origin of activity and stability enhancement for Ag_3_PO_4_ photocatalyst after calcination based on the systematical analyses of the structures, morphologies, chemical states of elements, oxygen defects, optical absorption properties, separation and transfer of photogenerated electron-hole pairs, and active species. The results indicate that oxygen vacancies (V_O_˙˙) are created and metallic silver nanoparticles (Ag NPs) are formed by the reaction of partial Ag^+^ in Ag_3_PO_4_ semiconductor with the thermally excited electrons from Ag_3_PO_4_ and then deposited on the surface of Ag_3_PO_4_ microspheres during the calcination process. Among the calcined Ag_3_PO_4_ samples, the Ag_3_PO_4_-200 sample exhibits the best photocatalytic activity and greatly enhanced photocatalytic stability for photodegradation of methylene blue (MB) solution under visible light irradiation. Oxygen vacancies play a significantly positive role in the enhancement of photocatalytic activity, while metallic Ag has a very important effect on improving the photocatalytic stability. Overall, the present work provides some powerful evidences and a deep understanding on the origin of activity and stability enhancement for the Ag_3_PO_4_ photocatalyst after calcination.

## 1. Introduction

Photocatalysis using semiconductors has attracted considerable attention due to its potential applications in solving energy supply and environmental pollution problems [1]. Much effort has been devoted to exploit new and efficient visible light-driven photocatalysts [2,3]. In particular, a breakthrough was made by Ye et al., who used silver phosphate (Ag_3_PO_4_) semiconductor as the visible light photocatalyst for the oxidation of water as well as the photodecomposition of organic compounds. The results indicated that Ag_3_PO_4_ semiconductor had an extremely higher visible light activity than commercial TiO_2−*x*_N*_x_* [4,5]. Zhu et al. investigated the origin of photocatalytic activation of Ag_3_PO_4_ via first-principle density functional theory, showing that Ag_3_PO_4_ had a large dispersion of conduction band (CB) and the inductive effect of PO_4_^3−^, favoring the separation of electron-hole pairs [6]. In the past several years, various strategies have been developed to further improve the photocatalytic activity and stability of Ag_3_PO_4_ under visible light irradiation. Coupling of Ag_3_PO_4_ with other semiconductors or noble metals is an effective approach to promote the charge separation efficiency of Ag_3_PO_4_, and thus improve the photocatalytic activity. Some coupled systems such as Ag_3_PO_4_/TiO_2_ [7,8,9], Ag_3_PO_4_/AgX (X = Cl, Br, I) [10], Ag_3_PO_4_/Ag [11,12,13], Ag_3_PO_4_/g-C_3_N_4_ [14,15] and Ag_3_PO_4_/graphene [16,17,18,19] have recently been developed to improve the photocatalytic activity of Ag_3_PO_4_. On the other hand, morphological engineering design is another effective approach to modify the photocatalytic activity of Ag_3_PO_4_. For instance, our research group synthesized Ag_3_PO_4_ crystals with various morphologies such as branch, tetrapod, nanorod, and triangular prism [20] and tetrahedral Ag_3_PO_4_ mesocrystals [21]. Additionally, other groups also prepared cubic sub-microcrystals [22], colloidal nanocrystals [23], hierarchical porous microcubes [24], tetrapod microcrystals [25,26,27], and two-dimensional dendritic nanostructures [28] of Ag_3_PO_4_. Nevertheless, researchers are still trying their best to explore novel and facile ways to further improve the photocatalytic activity and stability of Ag_3_PO_4_.

Recently, we found that the photocatalytic activity of Ag_3_PO_4_ was highly enhanced after calcination process in the air. However, we do not want to avoid the fact that two papers were recently published (nearly) simultaneously. Chong et al. prepared Ag_3_PO_4_ by using AgNO_3_ as the precursor, and found that Ag_3_PO_4_ annealed at 400 °C showed the best photocatalytic degradation performance under visible light due to the synergistic effect of crystallinity, oxygen vacancies and specific surface [29]. Nevertheless, it lacked the direct evidence for the formation of oxygen vacancies as well as the content of oxygen vacancies, and the detailed photocatalytic mechanism was not discussed. On the contrary, Yan et al. reported that the thermal treatment yielded many Ag vacancies within Ag_3_PO_4_ lattices, and both metallic Ag NPs and Ag vacancies in annealed Ag_3_PO_4_ samples were beneficial to the enhancement of photocatalytic activity toward methyl orange (MO) degradation under visible light irradiation [30]. However, no corresponding direct evidence was introduced to prove the presence of Ag vacancies within Ag_3_PO_4_ lattices. Therefore, some interesting questions naturally arise: What makes the contrary behavior? What are the substantial changes of Ag_3_PO_4_ during the calcination process? What is the evidence?

In this work, the substantial changes happened to Ag_3_PO_4_ after calcination were investigated based on the systematical analyses of the structures, morphologies, chemical states of elements, oxygen defects (including the direct and indirect evidences for the formation of oxygen vacancies and the content of oxygen vacancies), optical absorption properties, separation and transfer of photogenerated electron-hole pairs, and active species. The reasons for the enhancement of photocatalytic activity and stability of calcined Ag_3_PO_4_ series samples were analyzed and revealed. Furthermore, the transfer pathways of photo-induced carriers and degradation mechanism were proposed and discussed in detail.

## 2. Experimental

### 2.1. Synthesis

The calcined Ag_3_PO_4_ photocatalysts were synthesized as follows. Firstly, the pristine Ag_3_PO_4_ sample was prepared by a co-precipitation method in the dark at room temperature, according to a procedure described in the literature [4]. Typically, 1 g Na_2_HPO_4_ was dissolved into 250 mL deionized water to form a transparent solution. Then, 13.5 mL CH_3_COOAg solution (0.15 M) was dropped into the above Na_2_HPO_4_ solution under stirring. After stirring for 0.5 h, the obtained sample was filtered, washed with deionized water and ethanol, and dried at 80 °C overnight in vacuum. Secondly, the as-obtained Ag_3_PO_4_ precursor was placed in quartz boat and calcined in muffle furnace at a heating rate of 1 °C/min for 1 h at 100, 200, 300, and 400 °C in air atmosphere, respectively. The final Ag_3_PO_4_ photocatalysts were denoted as Ag_3_PO_4_-T, where T refers to the calcination temperature (100, 200, 300, and 400 °C).

To identify the roles of metallic Ag and oxygen vacancies, Ag/Ag_3_PO_4_ composite was prepared by photodeposited method using the pristine Ag_3_PO_4_ as the precursor. The synthesis details are as follows. The as-obtained Ag_3_PO_4_ precursor was dispersed into the CH_3_COOAg solution and irradiated with a 300 W Xe lamp for 1 h. Then the photocatalyst was filtered, washed with deionized water and ethanol, and dried at 80 °C overnight, which was marked as Ag/Ag_3_PO_4_-PD.

### 2.2. Characterization

The phase of the as-prepared samples was identified using Rigaku D/max-2400 X-ray diffractometer (XRD, Rigaku, Tokyo, Japan) with Ni-filtered Cu Kα radiation at room temperature. Thermogravimetric and differential scanning calorimetric analyses (TG-DSC) were performed with a NETZSCH DSC 200 F3 under an air flow, and the measurements were conducted from room temperature to 600 °C at a heating rate of 10 °C/min. Brunauer-Emmett-Teller (BET) surface area measurements were carried out using a Micromeritics ASAP 2000 system (Micromeritics Instrument Corp., Atlanta, GA, USA). The morphology was investigated by scanning electron microscopy (SEM, JSM-7500F, JEOL, Tokyo, Japan). X-ray photoelectron spectroscopy (XPS) measurements were carried out to investigate the surface chemical compositions and states with Al Kα X-ray (*hν* = 1486.6 eV) radiation source (K-Alpha, Thermo Electron, Waltham, MA, USA). The binding energies (BE) were referenced to the adventitious C 1s peak (284.6 eV) which was used as an internal standard to take into account charging effects. Electron paramagnetic resonance (EPR) spectra were recorded on a JEOL JES-FA200 EPR spectrometer (JEOL, Tokyo, Japan) at room temperature. A combination of Gaussian and Lorentzian functions was used to fit the curves. Photoluminescence (PL) spectra were recorded on a 48000DSCF luminescence spectrometer (SLM Corp., Edison, NJ, USA) at room temperature by using a continuous-wave 325 nm He-Cd laser as the excitation source. UV-Vis diffuse reflectance spectra (DRS) were obtained on a UV-Vis spectrophotometer (UV-2600, Shimadzu, Kyoto, Japan) using BaSO_4_ as reference. The samples were pressed into a thin disk and fixed in a homemade quartz cell. The electrochemical impedance spectra (EIS) and Mott-Schottky (MS) plots of the as-prepared photocatalysts were measured on an electrochemical analyzer (CHI660E, Shanghai Chenhua Instruments Co., Ltd., Shanghai, China), and the details are described in Supplementary Materials.

### 2.3. Photocatalytic Tests

According to the test method of photocatalytic materials for purification of water solution (GB/T 23762-2009, China) [31], the visible light photocatalytic activity of as-prepared samples was evaluated by degradation of MB in aqueous solution. The test details are as follows. Firstly, 0.10 g of catalyst was suspended in an MB aqueous solution (200 mL, 10 mg/L) in the dark for 1 h to gain the adsorption-desorption equilibrium. After the adsorption equilibrium of MB, the photocatalytic degradation reaction was carried out in a beaker with a circulating water system to remove the thermal effect of light. Light from a 300 W Xe lamp (HSX-UV300, Beijing NBET Technology Co. Ltd., Beijing, China), passed through a UV light filter film (to remove radiation with λ < 420 nm), was focused onto the reaction cell. When the light was turned on, at given time intervals, approximately 4 mL of the reaction suspension was sampled and thoroughly separated by means of high-speed centrifugation (18,000 rpm for 10 min) to reduce the experimental errors as much as possible. Then, clear and transparent filtrate was analyzed by recording the maximum absorbance at 664 nm in the UV-visible spectrum of MB. The degradation efficiency at time *t* was determined from the value of *C_t_*/*C*_0_, where *C*_0_ is the initial concentration and *C_t_* is the concentration of MB at time *t*.

The photocatalytic stability of pristine Ag_3_PO_4_ and Ag_3_PO_4_-200 was also investigated by checking the cycling runs in photocatalytic degradation of MB. Four consecutive cycles were completed and each cycle lasted for 14 min. After each cycle, the photocatalyst was filtrated and washed thoroughly with deionized water, and then fresh MB solution (10 mg/L) was added again for the next cycling run. The Ag_3_PO_4_-200 after second cycle was marked as the used Ag_3_PO_4_-200 sample.

The active species capturing experiments were carried out to study the photocatalytic mechanism. Different radical scavengers such as ethylenediaminetetraacetic acid disodium salt (EDTA-2Na, 1 mmol/L), tert-butyl alcohol (t-BuOH, 1 mmol/L) and 1,4-benzoquinone (BQ, 1 mmol/L) were added to the MB aqueous solution, sequentially, to capture various active species. Then, the remaining experimental processes were similar to the above photocatalytic activity test.

## 3. Results and Discussion

### 3.1. Phenomenon of the Enhanced Photocatalytic Activity and Stability

**Figure 1:**
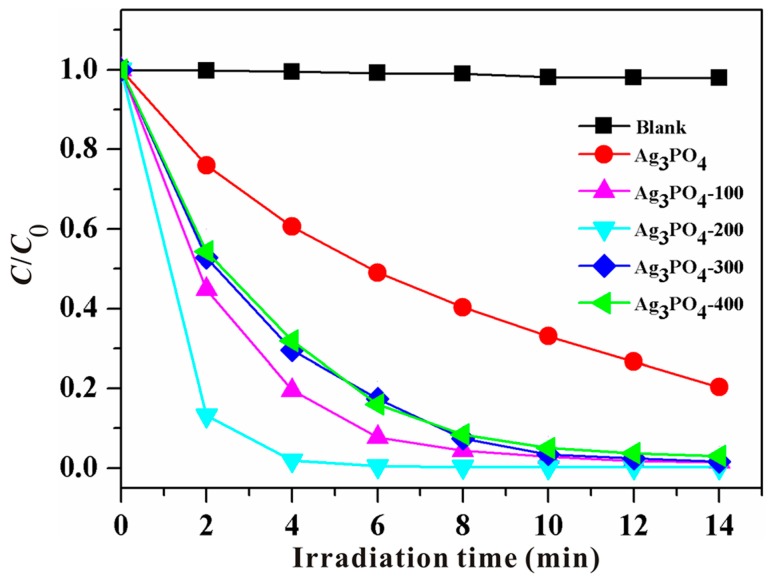
Photocatalytic degradation of MB over pristine Ag_3_PO_4_ and Ag_3_PO_4_-T (T = 100, 200, 300 and 400) samples under visible light irradiation.

Figure 1 shows the photocatalytic activities of pristine Ag_3_PO_4_ and Ag_3_PO_4_-T (T = 100, 200, 300 and 400) under visible light irradiation. First of all, MB solution was illuminated under visible light irradiation in the absence of photocatalyst and the result indicates that the self-degradation of MB is neglectable. The photocatalytic degradation efficiency of MB over pristine Ag_3_PO_4_ and Ag_3_PO_4_-T follows the order of Ag_3_PO_4_-200 > Ag_3_PO_4_-100 > Ag_3_PO_4_-300 ≈ Ag_3_PO_4_-400 > pristine Ag_3_PO_4_. The variation of absorption intensity of MB dye solutions over these samples at different irradiation times was recorded (Supplementary Materials Figure S1), which strongly supports the above result. Clearly, the photocatalytic activity of calcined Ag_3_PO_4_ samples is much higher than that of pristine Ag_3_PO_4_. More strikingly, the Ag_3_PO_4_-200 sample shows the highest photocatalytic activity among these calcined Ag_3_PO_4_ series samples, with the decomposition of about 98% MB within 4 min, while only 40% MB was decomposed by pristine Ag_3_PO_4_ within 4 min, even, only 80% MB was decomposed by pristine Ag_3_PO_4_ after 14 min under visible light irradiation. It is obvious that the photocatalytic activity of Ag_3_PO_4_ was significantly enhanced after calcination. This enhancement strategy aroused our great interest, which requires neither the combination with other semiconductor nor the design of morphology engineering. Anyway, this phenomenon of the enhanced photocatalytic activity suggests that something happened to Ag_3_PO_4_ during the simple calcination process.

**Figure 2:**
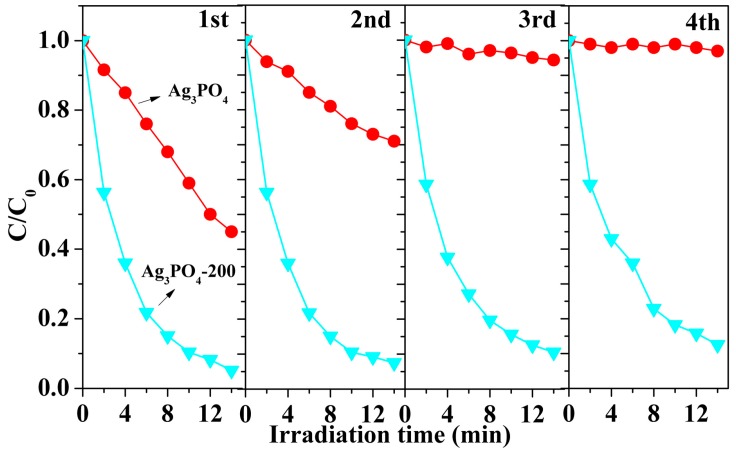
Cycling curves of photocatalytic degradation of MB solution in the presence of pristine Ag_3_PO_4_ and Ag_3_PO_4_-200, respectively.

To investigate the photocatalytic stability, pristine Ag_3_PO_4_ and Ag_3_PO_4_-200 were selected and used to carry out the cycling runs in photocatalytic degradation of MB under visible light irradiation, as displayed in Figure 2. It is observed that the Ag_3_PO_4_-200 still maintains a high photocatalytic activity even after four cycles. In contrast, the pristine Ag_3_PO_4_ shows a significant decrease in its photocatalytic degradation efficiency, and almost no MB degradation occurs over pristine Ag_3_PO_4_ after four cycles. This result clearly indicates that the Ag_3_PO_4_-200 sample possesses excellent photocatalytic stability.

### 3.2. Investigation of Substantial Changes

In order to make clear the origin of the enhanced photocatalytic activity and stability for Ag_3_PO_4_ after calcination, XRD, TG-DSC, BET, SEM, XPS, EPR, PL and UV-Vis DRS technologies were employed.

**Figure 3:**
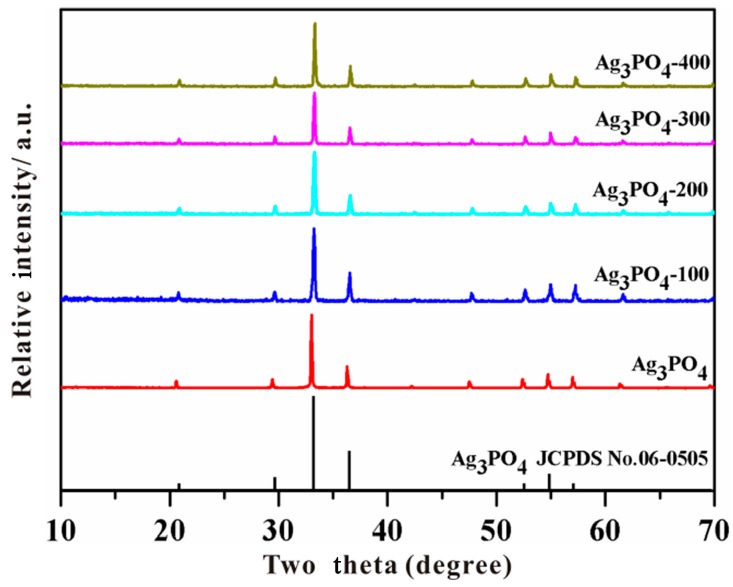
XRD patterns of pristine Ag_3_PO_4_ and Ag_3_PO_4_-T (T = 100, 200, 300 and 400) (the standard data for body-centered cubic Ag_3_PO_4_ (JCPDS No. 06-0505) are also presented at the bottom for comparison).

It is well-known that a high calcination temperature always improves the crystallization of photocatalysts [32]. In order to examine whether the crystallinity is enhanced after calcination process, the as-prepared samples were identified by XRD. As shown in Figure 3, the XRD pattern of the as-prepared pristine Ag_3_PO_4_ is in good accordance with the standard data (JCPDS No. 06-0505), indicating the formation of the pure Ag_3_PO_4_ phase with a body-centered cubic (bcc) structure. The intense and well-defined diffraction peaks suggest that pristine Ag_3_PO_4_ sample is well crystallized. After calcination in air, the crystal structure of the resulted samples is retained, showing a good thermal stability of the pristine Ag_3_PO_4_. However, it should be noted that the peak intensity of Ag_3_PO_4_-T samples does not obviously increase as expected. Especially, the samples of Ag_3_PO_4_-200, Ag_3_PO_4_-300 and Ag_3_PO_4_-400 show slightly decreased peak intensities, meaning that the crystallinity of these samples is slightly reduced.

Figure S2 presents TG-DSC curves of pristine Ag_3_PO_4_ measured in air atmosphere. The first weight loss below 150 °C is attributed to the release of free water (ca. 0.7 wt %). The second weight loss (ca. 1.2 wt %) ranging from 150 to 371 °C is due to the dehydration of pristine Ag_3_PO_4_. A sharp endothermic peak at 528 °C is observed in the DSC curve, which can be attributed to the melting of Ag_3_PO_4_ instead of the phase change [33]. This result is consistent with the XRD results.

BET surface areas of as-prepared Ag_3_PO_4_ samples were measured to estimate the influence of the surface area on the photocatalytic activity. However, it is found that the BET surface areas of pristine Ag_3_PO_4_, Ag_3_PO_4_-100, Ag_3_PO_4_-200, Ag_3_PO_4_-300, and Ag_3_PO_4_-400 are relatively low, which are 1.02, 0.83, 0.52, 0.31, and 0.16 m^2^/g, respectively. It is clear that the BET surface area decreases as the calcination temperature rises. Therefore, it is considered that the BET surface area is not a positive factor for the enhanced activity of calcined Ag_3_PO_4_ samples.

**Figure 4:**
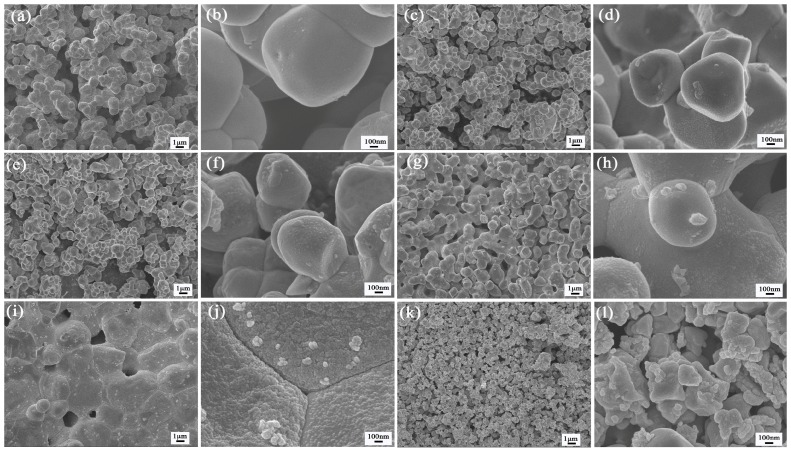
SEM images of: pristine Ag_3_PO_4_ (**a**,**b**); Ag_3_PO_4_-100 (**c**,**d**); Ag_3_PO_4_-200 (**e**,**f**); Ag_3_PO_4_-300 (**g**,**h**); Ag_3_PO_4_-400 (**i**,**j**); and the reused Ag_3_PO_4_-200 sample (**k**,**l**).

Figure 4a–j shows SEM images of the pristine and calcined Ag_3_PO_4_ samples. As shown in Figure 4a,b, the pristine Ag_3_PO_4_ consists of spherical particles with an estimated average diameter of ~0.8–1.2 μm. Compared with pristine Ag_3_PO_4_, it can be clearly visible that some nanoparticles with size of ~5–10 nm are adorned on the surface of Ag_3_PO_4_ microspheres (Figure 4c,d) for Ag_3_PO_4_-100. A similar structure is maintained in the samples of Ag_3_PO_4_-200 and Ag_3_PO_4_-300. Moreover, it can be seen that the amount of nanoparticles on the surface increases as the calcination temperature rises, indicating that the thermal treatment promotes the grain growth. However, for Ag_3_PO_4_-400, large particles are linked end-to-end and thus self-assembled into a chain-structure as the higher temperature may promote the disorder movement of fine particles [34], and almost all the surface of Ag_3_PO_4_ is covered with sintered nanoparticles. Figure 4k,l shows the SEM images of the reused Ag_3_PO_4_-200 sample. It can be seen that the surface of Ag_3_PO_4_ particles becomes unshaped and rough, and the nanoparticles are assembled on the surface of Ag_3_PO_4_. In addition, the quantity of nanoparticles was not significantly increased compared with fresh Ag_3_PO_4_-200 sample (Figure 4e,f). These nanoparticles with a size of ~5–10 nm, however, are hard to probe by high resolution transmission electron microscopy (HRTEM) because the structure of Ag_3_PO_4_ materials could be destroyed by high-energy electron beams during measurements [20,35].

In order to make these nanoparticles clear, XPS spectra were employed to further analyze the surface structure and composition. It can be seen from Figure S3 that the XPS survey spectra with peak identification for all samples are similar, which contain the elements of C, O, Ag, and P, and no obvious impurities can be detected. The C peak is attributed to adventitious hydrocarbon from the XPS instrument.

**Figure 5:**
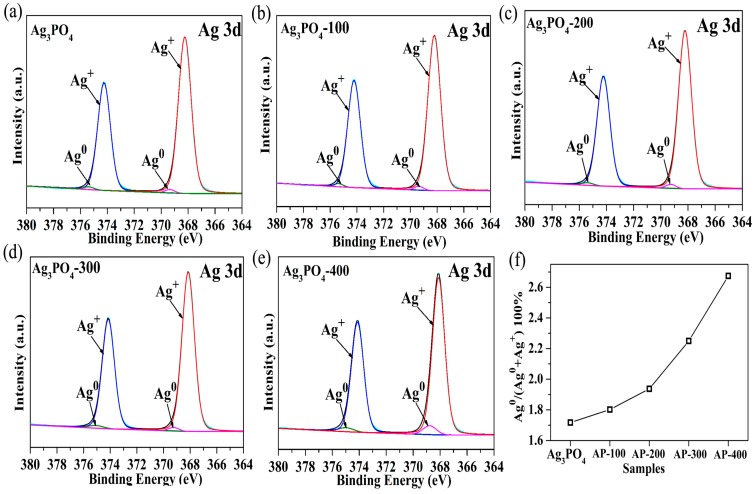
High-resolution XPS spectra of Ag 3d region of pristine Ag_3_PO_4_ (**a**); and Ag_3_PO_4_-T (T = 100, 200, 300 and 400) (**b**–**e**); and the ratios of peak area attributed to Ag^0^ to the total area of all Ag3d peaks for these samples (**f**) (AP denotes Ag_3_PO_4_ in (**f**)).

As shown in Figure 5a–e, the high-resolution XPS spectra of Ag 3d region of the as-prepared samples consist of two individual peaks at ~374 and ~368 eV, which can be attributed to Ag 3d3/2 and Ag 3d5/2 binding energies, respectively [8,36]. Since the peaks are asymmetrical, the Ag 3d peaks are further divided into four different peaks at 374.2, 375.4, 368.2 and 369.4 eV, respectively [37]. The peaks at 375.4 and 369.4 eV can be ascribed to metal Ag^0^, while the signals at 368.2 and 374.2 eV are associated with Ag^+^ ions [37]. This result clearly indicates the presence of metallic Ag, but it could not eliminate the existence of Ag_2_O. Moreover, as shown in Figure 5f, it is observed that the peak area attributed to Ag^0^ is increased with the increase of calcination temperature. The surface Ag^0^ content for the pristine Ag_3_PO_4_, Ag_3_PO_4_-100, Ag_3_PO_4_-200, Ag_3_PO_4_-300, and Ag_3_PO_4_-400, is calculated to be about 1.7%, 1.8%, 1.9%, 2.2%, and 2.6%, respectively, indicating that the surface content of Ag^0^ increases with the increase of the calcination temperature, being accordance with SEM results. Furthermore, it should be mentioned that metallic Ag are not probed by XRD patterns because the amount of Ag^0^ in the calcined samples is below the detection limit (5 mol %) of the XRD analysis [38], as confirmed by the calculated result in Figure 5f. In addition, the reused Ag_3_PO_4_-200 sample was collected after a second run and characterized by XPS technique. In Figure S4a, it can be seen that the Ag 3d peaks are further divided into four different peaks at 374.2, 375.4, 368.2 and 369.4 eV, respectively. The peaks at 375.4 and 369.4 eV can be ascribed to metal Ag^0^ [37], indicating the presence of metal Ag in the reused Ag_3_PO_4_-200 sample. Moreover, the Ag^0^ content for the reused Ag_3_PO_4_-200 sample was calculated to be about 2.08%, which is close to that for fresh Ag_3_PO_4_-200 sample (1.9%), as shown in Figure S4b. This result indicates that there is no obvious photocorrosion phenomenon for the fresh Ag_3_PO_4_-200 sample during photocatalytic process.

**Figure 6:**
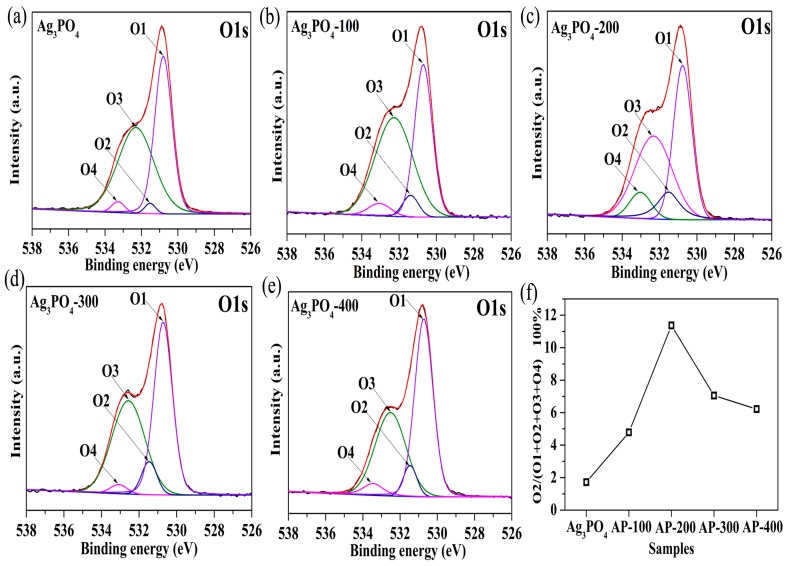
High-resolution XPS spectra of O1s region of pristine Ag_3_PO_4_ (**a**); and Ag_3_PO_4_-T (T = 100, 200, 300 and 400) (**b**–**e**); and the ratios of the O2 peak area to the total area of all O1s peaks for these samples (**f**) (AP denotes Ag_3_PO_4_ in (**f**)).

Figure 6a–e shows the O1s peaks of pristine Ag_3_PO_4_ and Ag_3_PO_4_-T (T = 100, 200, 300 and 400). The O1s peaks of all samples are divided into four different peaks at 530.8 ± 0.1 (O1 peak), 531.5 ± 0.1 (O2 peak), 532.3 ± 0.1 (O3 peak) and 533.2 ± 0.1 eV (O4 peak), respectively. The O1 peak can be attributed to the non-bridging (P=O) oxygen atoms [39,40]. The O2 peak is associated with oxygen vacancies or defects within the matrix, which are originally occupied by O^2−^ ions [41,42,43,44], and the content of oxygen vacancies or defects depends on the intensity or the area of this peak [42]. In addition, it was reported that the peak attributed to O^2−^ in Ag_2_O was located at 528.8 eV [45], which is much lower than the value of O2 peak (531.5 ± 0.1 eV) in our study. Then, it can be concluded that O^2−^ is due to oxygen vacancies and not to the formation of Ag_2_O. O3 and O4 peaks at higher binding energies can be assigned to the bridging oxygen atoms (P–O–Ag) in the structure of Ag_3_PO_4_ and the specific absorbed oxygen, such as adsorbed oxygen and water molecules, respectively [46]. Additionally, as shown in Figure 6f, the ratio of the O2 peak area to the total area of all O1s peaks (O1 + O2 + O3 + O4) first rapidly increases with the increase of the calcination temperature, and the ratio for Ag_3_PO_4_-200 reaches maximum of 11.4%, which is circa six times higher than that of the pristine Ag_3_PO_4_ sample (1.8%). This indicates that Ag_3_PO_4_-200 has the highest density related to deficient oxygen, which is attributed to the release of oxygen from Ag_3_PO_4_ during the calcination process, giving rise to the formation of V_O_˙˙ on the surface of Ag_3_PO_4_ [29]. V_O_˙˙ could create shallow donor states below CB and increase the electron concentration, thus leading to the enhancement of the electrical conductivity and the increase of active sites [47].

Given the SEM results and the analyses of high-resolution XPS spectra of Ag 3d and O1s regions of pristine Ag_3_PO_4_ and Ag_3_PO_4_-T (T = 100, 200, 300 and 400), it is reasonable to assume that Ag (NPs) are deposited on the surface of Ag_3_PO_4_ microspheres. Moreover, it is obvious that metal Ag^0^ exists in the as-prepared Ag_3_PO_4_ samples including the pristine Ag_3_PO_4_, which might result from the “self-corrosion” of pristine Ag_3_PO_4_ during the period of preservation in the dark [48]. Nevertheless, the fact that the surface Ag^0^ content increases with the increase of the calcination temperature could originate from the thermally decomposition during calcination process. During the heat treatment, Ag_3_PO_4_ semiconductor is activated at high temperatures, and thus electrons are excited from the valence band (VB) into the CB of Ag_3_PO_4_, leaving holes behind. In our work, because CH_3_COOAg was used as the raw material, the residual CH_3_COO^−^ species on the Ag_3_PO_4_ surface may capture and react with thermally excited holes, which will inhibit the recombination of thermally excited charge carriers and further promote the reduction reaction between electrons and Ag^+^ ions within Ag_3_PO_4_ matrix, resulting in the formation of metallic Ag in the air [30]. Simultaneously, molecular O_2_ in air condition adsorbed on Ag_3_PO_4_ can also act as an electron scavenger to trap the thermally excited electrons to form the superoxide radicals (•O_2_^−^) active specie, which is the predominant reaction [30]. However, under the oxygen-free condition, the thermally excited electrons should mainly participate in the reduction of Ag^+^ ions into metallic Ag; thus, compared with that annealed in the air condition, the calcined samples should have higher content of metallic Ag [30]. Anyway, metallic Ag was also formed in the presence of air.

**Figure 7:**
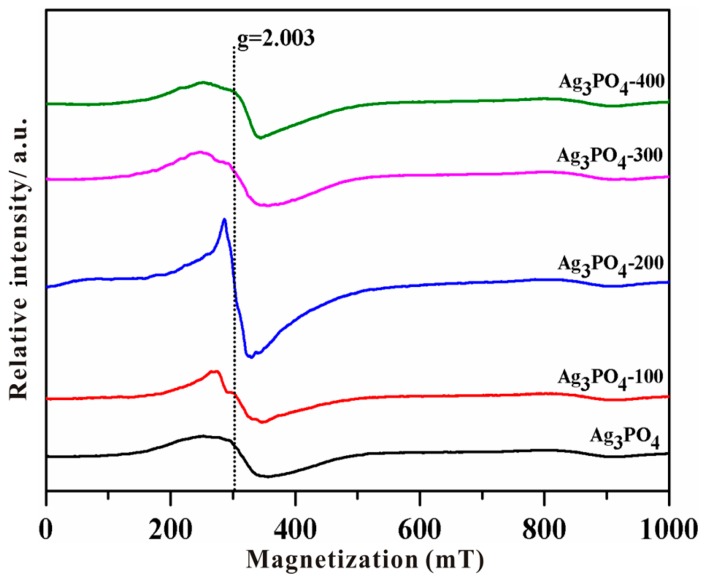
EPR spectra of pristine Ag_3_PO_4_ and Ag_3_PO_4_-T (T = 100, 200, 300 and 400) recorded at room-temperature.

EPR is considered to be a sensitive and direct method to monitor the behaviors of oxygen defects [48]. The EPR spectra of the pristine and calcined Ag_3_PO_4_ samples are displayed in Figure 7. The peak at *g* = 2.001–2.004 has been reported previously and it can be attributed to natural surface oxygen vacancies [49,50]. Notably, Ag_3_PO_4_-200 has much higher intensity of the EPR signal at *g* factor of ~2.003 for the oxygen vacancy than both pristine Ag_3_PO_4_ and other calcined Ag_3_PO_4_ samples, implying the increase of oxygen vacancies in the sample of Ag_3_PO_4_-200, which is consistent with the O1s XPS results.

**Figure 8:**
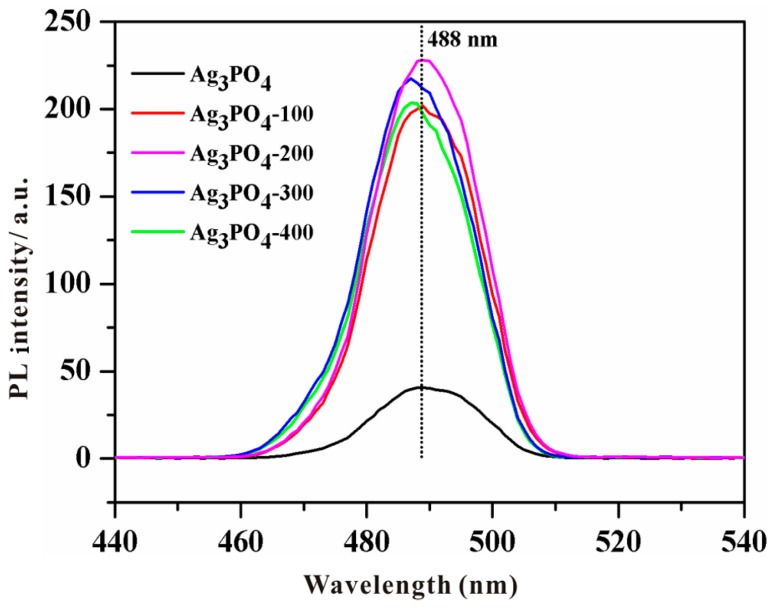
PL spectra of the as-prepared samples (excitation wavelength: 325 nm).

It is well-known that the band-band PL signals of semiconductor materials are caused by the recombination of photoinduced charge carriers. In general, a lower intensity of band-band PL signals indicates a decrease in the recombination rate of photogenerated charge carriers [51]. However, the excitonic PL signals mainly result from surface oxygen vacancies and defects of semiconductors [52], because the oxygen vacancies as well as defects can easily bind photo-induced electrons to form excitons and the exciton energy level can be formed near the bottom of the CB so that PL signal can easily occur. The larger the content of oxygen vacancy or defect, the stronger the PL signal [52]. As shown in Figure 8, the strong emission peak is located at about 488 nm for the as-prepared samples, which could be attributed to the band edge binding excitons resulting from the surface oxygen vacancies and defects [53]. In addition, the band-band PL peak at about 530 nm (corresponding to the band gap of ~2.4 eV for Ag_3_PO_4_ semiconductor) cannot be found [4]. Furthermore, the intensity of excitonic PL signal for the as-prepared samples follows the order of Ag_3_PO_4_-200 > Ag_3_PO_4_-300 > Ag_3_PO_4_-400 > Ag_3_PO_4_-100 > Ag_3_PO_4_ sample. This order is consistent with the content of oxygen vacancies obtained from the O1s XPS spectra. The result implies that the stronger the PL signal, the larger the surface oxygen vacancy concentration. Additionally, it can be concluded that the PL spectra provide an indirect evidence for the formation of oxygen vacancies.

**Figure 9:**
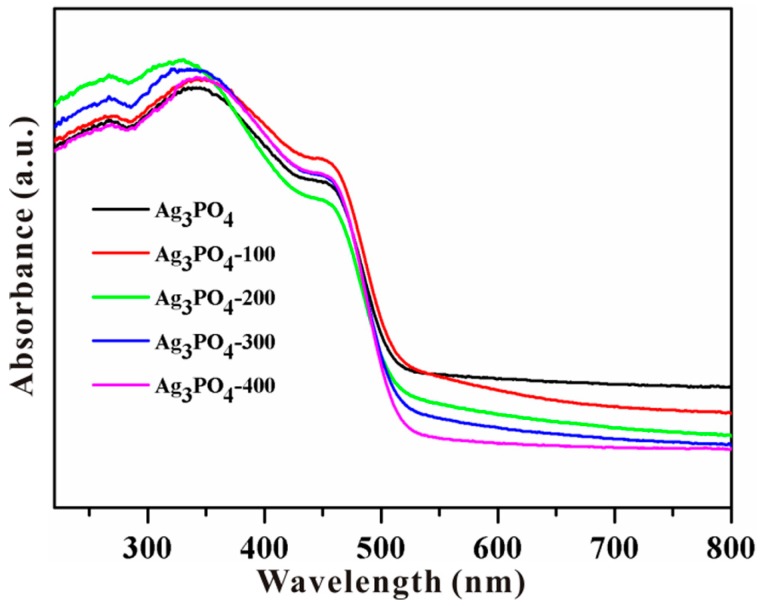
UV-Vis DRS of pristine Ag_3_PO_4_ and Ag_3_PO_4_-T (T = 100, 200, 300 and 400).

Figure 9 shows UV-visible DRS of pristine Ag_3_PO_4_ and Ag_3_PO_4_-T (T = 100, 200, 300 and 400). It can be observed that all Ag_3_PO_4_ samples show similar absorption plot. The pristine Ag_3_PO_4_ has a broader absorption in the visible region with an absorption edge at ~530 nm, which is attributed to the charge transfer response of Ag_3_PO_4_ from VB to CB. As seen from Figure S5, the estimated indirect band gap of pristine Ag_3_PO_4_ is 2.44 eV, and the thermal treatment decreased the energy gap of Ag_3_PO_4_-T (T = 100, 200, 300 and 400) series sample from 2.44 eV to 2.41 eV, resulting in an expanded light response. The band gap narrowing upon calcination may be caused by the formation of oxygen vacancies, which create a local state below CB of Ag_3_PO_4_. In addition, the localized surface plasmon resonance (LSPR) of metallic Ag is not observed. It might result from the fact that the metallic Ag^0^ content in the pristine Ag_3_PO_4_ and Ag_3_PO_4_-T (T = 100, 200, 300 and 400) samples is very low according to the calculation from high-resolution XPS spectra of Ag 3d region of these samples (Figure 5f). The metallic Ag^0^ with quite low content will not exhibit the plasmon absorption in the UV-Vis spectra. Similar phenomenon was also reported by Bi et al. [13], who synthesized the Ag/Ag_3_PO_4_ necklace-like heterostructure and did not observe the plasmon absorption in the UV-Vis spectra.

Based on the above analyses of O1s XPS spectra, EPR, PL and DRS, it is clear that oxygen vacancies are generated during the calcination process, and the Ag_3_PO_4_-200 sample shows the highest content of oxygen vacancies.

### 3.3. Mechanism of the Photocatalytic Reaction

**Figure 10:**
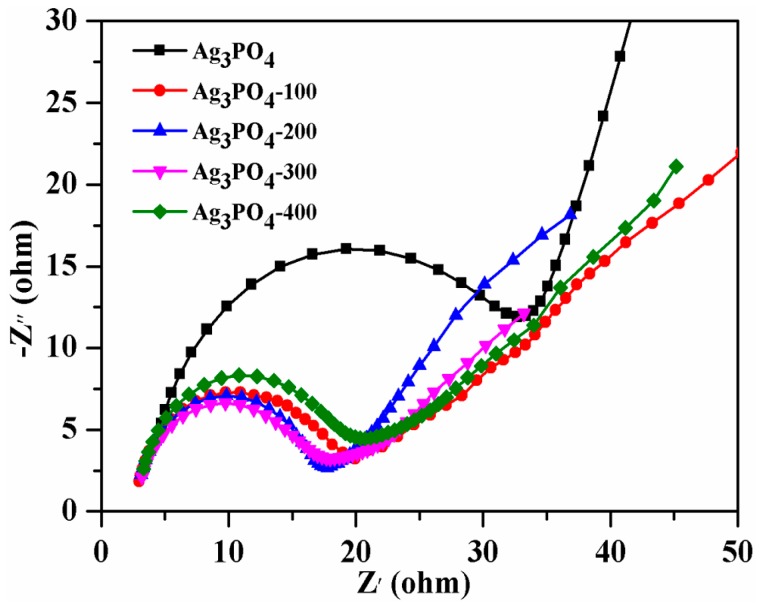
EIS Nyquist plots of the electrodes prepared by pristine and calcined Ag_3_PO_4_ samples under visible light irradiation.

EIS analysis is a powerful tool to investigate the transfer process of the photogenerated charges. Figure 10 displays EIS Nyquist plots of pristine and calcined Ag_3_PO_4_ samples. In Nyquist plots, the semicircle or the arch at high frequency represents the charge transfer process, and the diameter of the semicircle or the arch reflects the mobility of charges; that is, the smaller the diameter of the semicircle, the faster the electrons transfer [54]. Clearly, the diameters of the semicircle for the electrodes prepared by the calcined Ag_3_PO_4_ series samples are all much smaller than by the pristine Ag_3_PO_4_, indicating that the calcination process can enhance the electron mobility. As a result, the recombination of electron-hole pairs can be prevented to a certain extent. Among the electrodes prepared by the calcined Ag_3_PO_4_ series samples, the Ag_3_PO_4_-200 electrode shows the smallest semicircle, suggesting that Ag_3_PO_4_-200 owns a more effective separation of photogenerated electron-hole pairs and a faster interfacial charge transfer. This result strongly supports the highest photocatalytic degradation efficiency of MB over Ag_3_PO_4_-200, as displayed in Figure 1.

**Figure 11:**
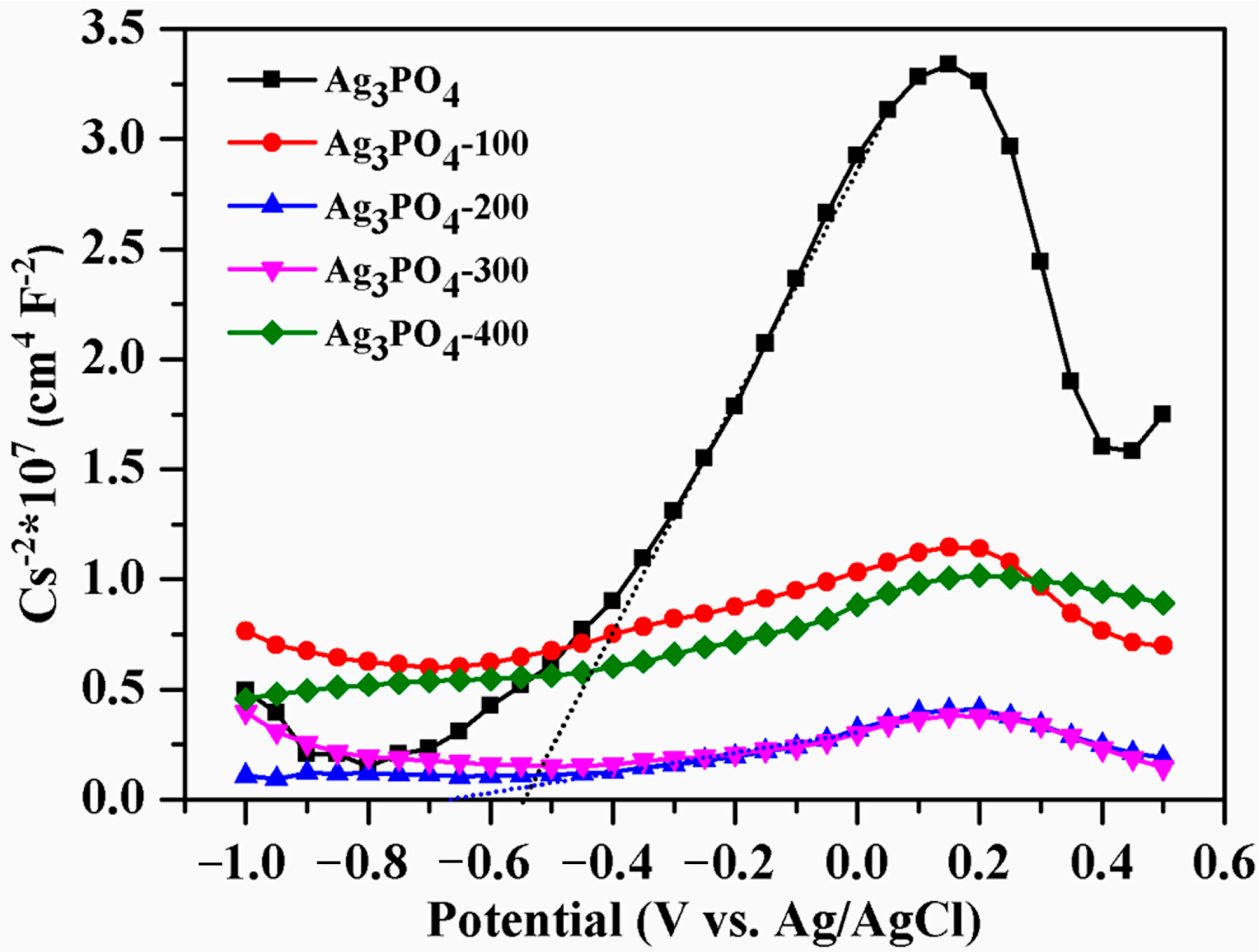
Mott-Schottky (MS) plots of the electrodes prepared by pristine and calcined Ag_3_PO_4_ samples.

Figure 11 shows Mott-Schottky plots for the electrodes prepared by pristine and calcined Ag_3_PO_4_ samples. All samples have a positive slope in the Mott-Schottky plots, as expected for the *n*-type semiconductors. Additionally, it is noteworthy that the slopes of the linear region for the calcined Ag_3_PO_4_ series samples are much smaller than that for pristine Ag_3_PO_4_, suggesting a higher donor density [55]. Generally, the higher the donor density, the faster the photocatalytic degradation rate [56]. Therefore, the Mott-Schottky results further confirm that the calcination process greatly promotes the charge transfer, favoring significantly the improvement of the photocatalytic activity.

**Figure 12:**
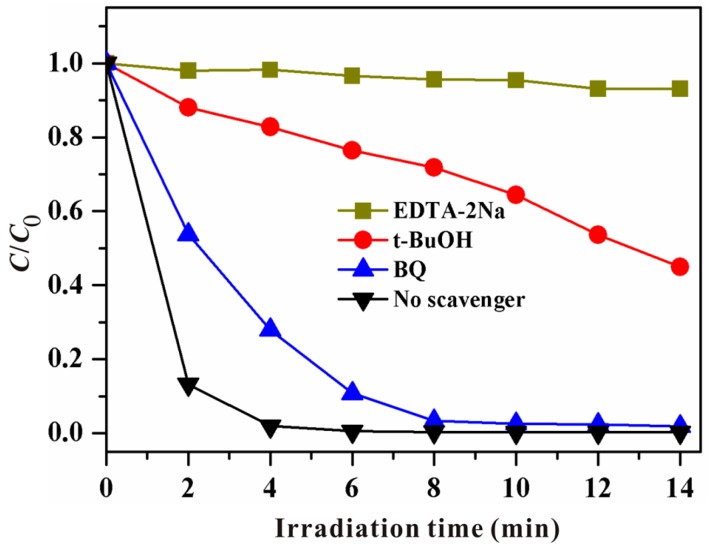
The photocatalytic activity of Ag_3_PO_4_-200 for the degradation of MB under visible light upon the addition of different scavengers.

A succession of control experiments was performed to understand the role of the photoexcited active species in the photocatalytic degradation of MB dye. Herein, EDTA-2Na [57], t-BuOH [58] and BQ [59] were used as scavengers of the trapped holes (h^+^), hydroxyl radical (•OH) and •O_2_^−^, respectively. Figure 12 presents the photocatalytic activity of Ag_3_PO_4_-200 upon the addition of different scavengers. The degradation efficiency greatly decreases to 6.9%, 55.1%, and 98.2% with the addition of EDTA-2Na, t-BuOH and BQ, respectively. Thus, the role of active species in the degradation of MB dye follows the order of h^+^ > •OH > •O_2_^−^. This result indicates that h^+^ and •OH are the most crucial species while there is little or no •O_2_^−^ during the photocatalytic degradation of MB in the Ag_3_PO_4_-200 system.

From the above analysis, it is clear that both metallic Ag and oxygen vacancies are present in the calcined Ag_3_PO_4_ samples. However, it is difficult to trace the beneficial effect back to either of them or both of them. To identify the roles of both species, the uncalcined Ag_3_PO_4_ was used as the precursor and metallic Ag was photodeposited on it non-thermally, marked as Ag/Ag_3_PO_4_-PD. Then, oxygen vacancies would likely not be formed.

**Figure 13:**
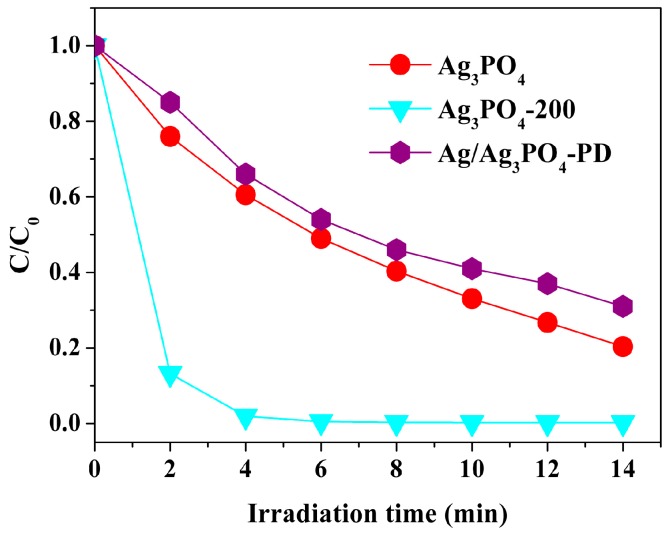
Photocatalytic degradation of MB over pristine Ag_3_PO_4_, Ag/Ag_3_PO_4_-PD, and Ag_3_PO_4_-200 under visible light irradiation.

Figure 13 shows a comparison of the photocatalytic activity over pristine Ag_3_PO_4_, Ag/Ag_3_PO_4_-PD, and Ag_3_PO_4_-200 samples under visible light irradiation. It is found that the photocatalytic degradation efficiency of Ag/Ag_3_PO_4_-PD is lower than that of pristine Ag_3_PO_4_ and Ag_3_PO_4_-200, indicating that metallic Ag has a negative effect on the photocatalytic activity. Thus, it can be concluded that oxygen vacancies play a significantly positive role in the enhancement of photocatalytic activity for the calcined Ag_3_PO_4_ samples. However, it should be pointed out that metallic Ag plays an important role in the enhancement of photocatalytic stability for the calcined Ag_3_PO_4_ samples because the metallic Ag can serve as a good electron acceptor for facilitating quick electron transfer from Ag_3_PO_4_ [13] as far as possible instead of remaining in the position of Ag^+^ ions in Ag_3_PO_4_ lattice, leading to the inhibition of photocorrosion of Ag_3_PO_4_ [11,12]. This explains why the Ag_3_PO_4_-200 sample exhibits improved photocatalytic stability.

**Scheme 1:**
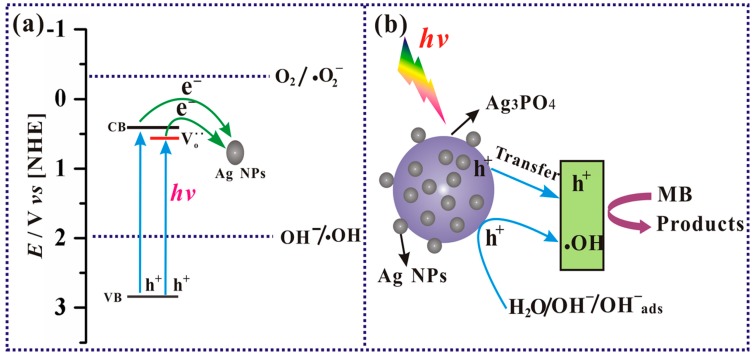
(**a**) Proposed transfer pathways of photoinduced carriers based on the energy levels of Ag_3_PO_4_ and oxygen vacancies; and (**b**) a schematic illustration of MB photodegradation for the calcined Ag_3_PO_4_ samples.

Since Ag_3_PO_4_ has a low CB level (+0.45 V vs. a normal hydrogen electrode (NHE)) [4,60], which is more positive than the potential for the single-electron reduction of oxygen (E(O_2_/•O_2_^−^) ≈ −0.33 V vs. NHE) [61,62], the electrons transferred to the surface of Ag NPs could not react with dissolved oxygen to generate the active groups of •O_2_^−^, but leaving a large number of h^+^ in the VB of Ag_3_PO_4_, as shown in Scheme 1a. This proposed mechanism is confirmed by the photogenerated carriers trapping experiment (Figure 12). The excess electrons on the surface of Ag NPs might react with oxygen molecules and H^+^ in the aqueous solution of MB to form H_2_O_2_ by multi-electron reduction process (O_2_ + 2H^+^ + 2e^−^ = H_2_O_2_ (aq), +0.682 V vs. NHE) [63].

Furthermore, the oxygen vacancies might form a local state below CB of Ag_3_PO_4_, leading to narrowing of band gap and thus the enhancement of the visible-light response (as confirmed by UV-Vis DRS). Once irradiated under visible light, a majority of electrons are able to jump from the VB to the local state formed by oxygen vacancies in Ag_3_PO_4_, and leave h^+^ in the VB. During the process of photocatalytic reaction, the formed oxygen vacancies and defects can become centers to capture photo-induced electrons, so the recombination of photo-induced electrons and holes can be effectively inhibited [64], leading to the enhancement of photocatalytic activity. Additionally, a large number of h^+^ with high oxidation potential (+3.0 V vs. NHE) [65] are produced and take part in the photocatalytic degradation reaction. This is consistent with the measured results of the mainly oxidative species. Of course, some of h^+^ can also react with OH^−^ group or H_2_O molecules to produce •OH radicals, which are responsible for the degradation of organic MB pollutant due to their high oxidation capacity, as illustrated in Scheme 1b.

## 4. Conclusions

The calcined Ag_3_PO_4_ photocatalysts with V_O_˙˙ have been successfully synthesized via a facile calcination method. During the calcination, Metallic Ag NPs are generated due to the reduction of Ag^+^ in Ag_3_PO_4_ semiconductor with the thermally excited electrons, and deposited on the surface of Ag_3_PO_4_ microspheres with the formation of the closely contacted interface between two components which is beneficial for the rapid transfer of the photo-excited electrons from Ag_3_PO_4_ to Ag NPs, and thus inhibiting the photocorrosion of Ag_3_PO_4_ that means the enhancement of photocatalytic stability. Notably, the Ag_3_PO_4_-200 sample owns the highest amount of V_O_˙˙, which could inhibit the recombination of photo-induced electron-hole pairs by acting as centers to capture photoinduced electrons and enhance the visible-light response by creating a shallow donor state below CB of Ag_3_PO_4_, leading to the enhancement of the electrical conductivity and the increase of active sites and then the highest photocatalytic activity. Moreover, it is found that a large number of h^+^ and •OH play a crucial role in the photocatalytic degradation of MB, while there is little or no •O_2_^−^ generated during the photocatalytic degradation of MB. In brief, the present work provides insight for the design of novel efficient visible-light photocatalysts.

## Supplementary Materials

The following are available online at www.mdpi.com/1996-1944/9/12/968/s1. Figure S1: UV-Vis absorption spectra of MB solutions separated from: pristine Ag_3_PO_4_ (a); Ag_3_PO_4_-100 (b); Ag_3_PO_4_-200 (c); Ag_3_PO_4_-300 (d); and Ag_3_PO_4_-400 (e) suspensions during illumination, Figure S2: TG-DSC curves of pristine Ag_3_PO_4_, Figure S3: The survey XPS spectra of pristine Ag_3_PO_4_ and Ag_3_PO_4_-T (T = 100, 200, 300 and 400), Figure S4: (a) High-resolution XPS spectrum of Ag 3d region of the reused Ag_3_PO_4_-200 sample; and (b) a comparison of Ag^0^ content for fresh Ag_3_PO_4_-200 sample and the reused Ag_3_PO_4_-200 sample, Figure S5: Plots of (*αhν*)^1/2^ versus photon energy (*hν*) of pristine Ag_3_PO_4_ (a); Ag_3_PO_4_-100 (b); Ag_3_PO_4_-200 (c); Ag_3_PO_4_-300 (d); and Ag_3_PO_4_-400 (e).
