# Supplementary material for: Origin of Activity and Stability Enhancement for Ag3PO4 Photocatalyst after Calcination
Source: Materials (Basel). 2016 Nov 29;9(12):968. doi: 10.3390/ma9120968 (PMC5457017; doi:10.3390/ma9120968)
Supplement: Supplementary file 1 [file materials-09-00968-s001.pdf]

Supplementary Materials: Origin of Activity and Stability Enhancement for Ag_3PO_4 Photocatalyst after Calcination

Pengyu Dong, Guihua Hou, Chao Liu, Xinjiang Zhang, Hao Tian, Fenghua Xu, Xinguo Xi and Rong Shao

Electrochemical Measurement

The electrochemical impedance spectra (EIS) and Mott-Schottky (MS) plots of the as-prepared photocatalysts were measured on an electrochemical analyzer (CHI660E) (Shanghai Chenhua Instruments Co., Ltd., Shanghai, China) in a standard three-compartment cell using 0.5 M Na_2SO_4 (pH = 6.8) solution as the electrolyte. For the preparation of working electrode for electrochemical measurements, a homogeneous catalyst ink was first prepared by dispersing 4 mg of catalyst material and 80 μL of a 5 wt % Nafion solution in 2 mL of H_2O by ultrasonication, and then 400 μL of catalyst ink dispersion was drop-coated directly onto the pre-cleaned indium tin oxide (ITO) glass surface by micro-syringe and placed on a hot plate to speed drying. The surface of working electrode exposed to the electrolyte was a circular film with the geometrical surface area of 4 cm^2. Platinum foil was used as counter electrode and Ag/AgCl electrode as the reference electrode. The EIS were measured at 0.0 V. A sinusoidal ac perturbation of 5 mV was applied to the electrode over the frequency range of 1–10^4 Hz. The MS plots were obtained at a frequency of 1 kHz.

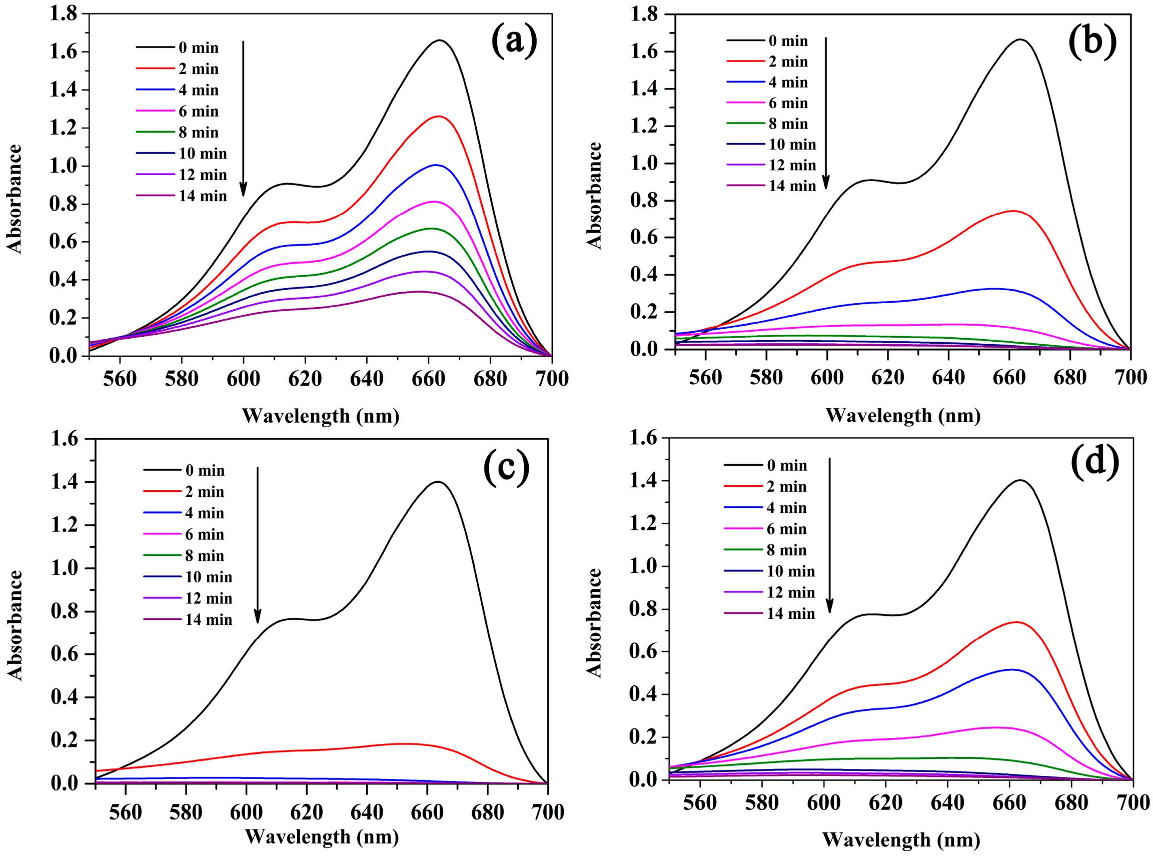

Figure S1. *Cont.*

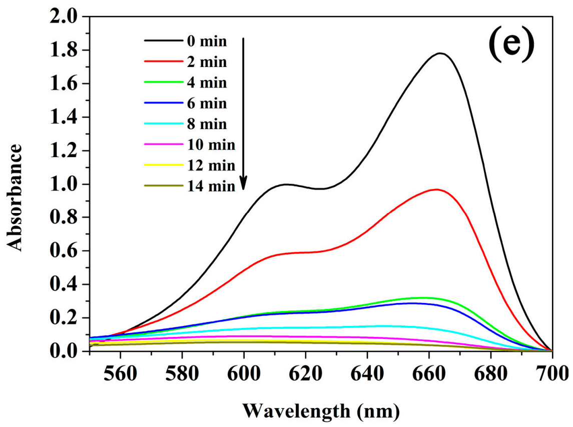

Figure S1. UV-Vis absorption spectra of MB solutions separated from: pristine Ag_3PO_4 (**a**); Ag_3PO_4-100 (**b**); Ag_3PO_4-200 (**c**); Ag_3PO_4-300 (**d**); and Ag_3PO_4-400 (**e**) suspensions during illumination.

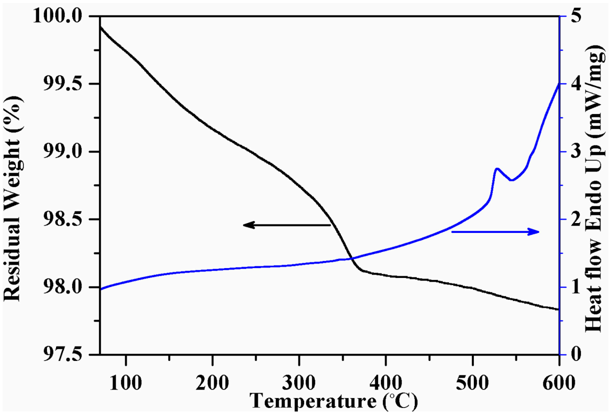

Figure S2. TG-DSC curves of pristine Ag_3PO_4.

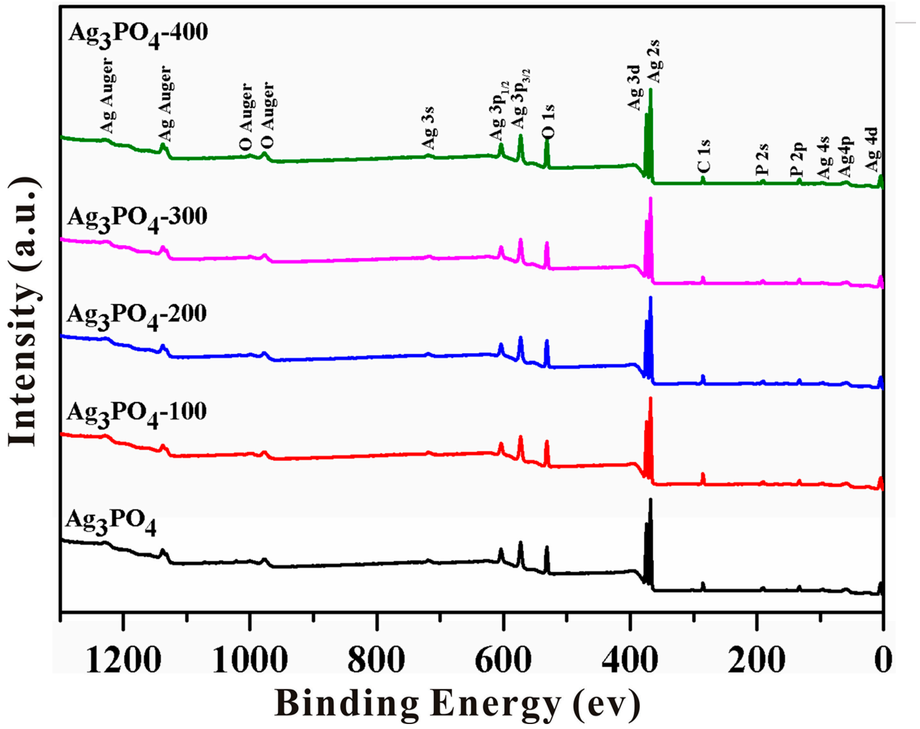

Figure S3. The survey XPS spectra of pristine Ag_3PO_4 and Ag_3PO_4-T (T = 100, 200, 300 and 400).

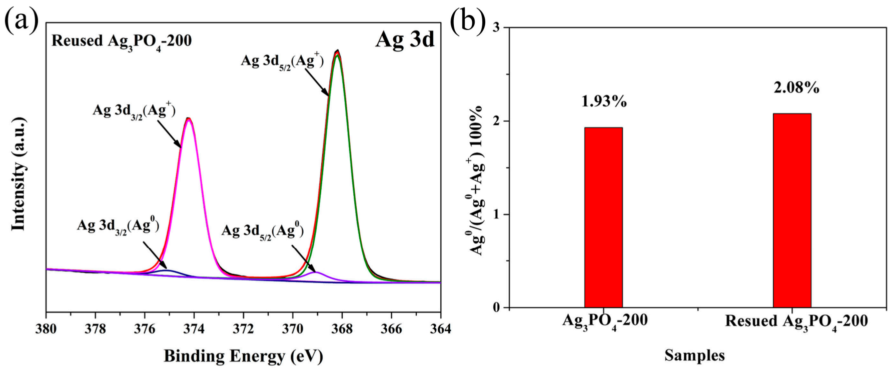

Figure S4. (**a**) High-resolution XPS spectrum of Ag 3d region of the reused Ag_3PO_4-200 sample; and (**b**) a comparison of Ag^0 content for fresh Ag_3PO_4-200 sample and the reused Ag_3PO_4-200 sample.

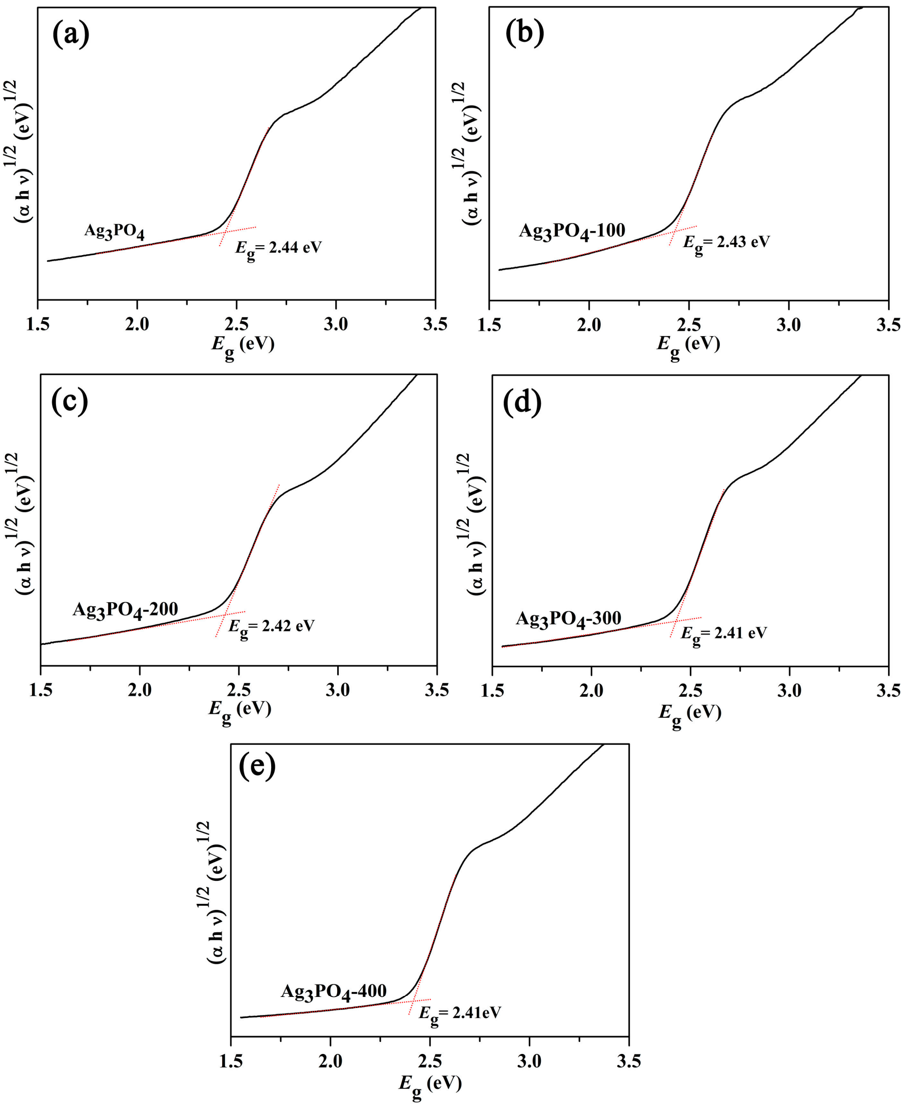

Figure S5. Plots of $(\alpha h\nu)^{1/2}$ versus photon energy ($h\nu$) of: pristine Ag_3PO_4 (**a**); Ag_3PO_4-100 (**b**); Ag_3PO_4-200 (**c**); Ag_3PO_4-300 (**d**); and Ag_3PO_4-400 (**e**).
